# American Veterinary College

**Published:** 1896-06

**Authors:** 


### AMERICAN VETERINARY COLLEGE.

The Twenty-first Annual Commencement held at Chickering Hall, Wednesday evening, March 25th, marked an important epoch in the history of veterinary colleges by this school having obtained her majority. The hall was well filled, and those who listened to the very witty and entertaining address of Prof. J. W. Dowling, M.D., spent one of the most pleasant hours of their lives.

The first event of the evening was the conferring of degrees upon the following-named happy graduates: Clarence Linwood Adams, Danielson, Ct.; Alfred Frederick Bollinger, Brooklyn, N. Y.; John Sutherland Buckley, Mt.

Washington, Md.; Samuel Sutherland Buckley, B.Sc., Mt. Washington, Md.; George Cohen, New York, N. Y.; Cyrus Harvey Du Bois, New Platz, N. Y.; John Daly Gogerty, New York, N. Y.; Robert Walter Grutzman, B.Sc., Strasburg, Germany; Leland Delano Ives, Wallingford, Ct.; Edward Nathaniel Leavy, New York, N. Y.; August Frederick Lange, San Antonio, Texas; Thomas Jewett Lee, East Granby, Ct.; Michael Joseph Murphy, Jr., New York, N. Y.; George W. Mead, Jr., Brooklyn, N. Y.; Harrison Price Monk, Brooklyn, N. Y.; Percival King Nichols, Port Richmond, N. Y.; Herbert William Pratt, Rome, N. Y.; Henry Wilson Peter, Saegersville, Pa.; John Passmore Rauch, Memphis, Tenn.; John Goddard Richardson, Portland, Me.; William R. Rosekrans, New York, N. Y.; Christian Rothaug, Brooklyn, N. Y.; Charles Henry Tilton, Jr., Ashland, Mass.; Robert Clarkson Vail, Rahway, N. J.; Charles Robert Witte, New Britain, Ct.; Frederick Adolph Zucker, New York, N. Y.

Next in order was the awarding of prizes, which was accompanied by a few pleasant remarks by Prof. L. H. Friedburg, Ph.D. The prizes and recipients were as follows: Trustees' Gold Medal for the best general examination, awarded to John S. Buckley, Mt. Washington, Md. The Faculty Gold Medal, for the best practical examination, awarded to Frederick A. Zucker, New York, N. Y. The Alumni Association Prize, second best general examination, awarded to Samuel S. Buckley, B.Sc., Mt. Washington, Md. Prize for the best paper presented and defended at the College Association, awarded to Herbert W. Pratt, Rome, N. Y. The Junior Anatomy Prize, silver medal, awarded to A. J. Pistor. A well-worded valedictory was very admirably read by Edward N. Leavy, D.V.S.

The music was one of the prominent, pleasant features of the evening. A little later on the members of the Faculty, Alumni, and invited guests sat down to an elaborate banquet at Clark's, and after coffee and segars listened to toast responses. Dr. Herbert Lowe, of Paterson, was speaker of the evening.

The exercises were conspicuous by the absence of Prof. Liautard and the presence of Prof. James L. Robertson, Dr. Dougherty, of Baltimore; Drs. Simon J. J. Harger, of the Veterinary Department of the University of Pennsylvania; W. Horace Hoskins, of Philadelphia, the President of the United States Veterinary Association, and H. D. Gill, Dean of the New York College of Veterinary Surgeons.

